# Editorial: Reaching to Grasp Cognition: Analyzing Motor Behavior to Investigate Social Interactions

**DOI:** 10.3389/fpsyg.2018.01236

**Published:** 2018-08-14

**Authors:** Claudia Gianelli, Maurizio Gentilucci

**Affiliations:** ^1^Universität Potsdam, Potsdam, Germany; ^2^Università degli Studi di Parma, Parma, Italy

**Keywords:** kinematics, social cognition, action observation, imitation, joint action, complementary actions, cooperation and competition, embodied cognition

## Introduction

Action planning and execution have always been fascinating topics for neuroscience and psychology. In particular, kinematics studies have contributed to shed light on how very basic actions (e.g., reaching-grasping) are affected by manipulating target properties, visually or linguistically presented stimuli and contextual information. Interestingly, recent studies have also shown how the social context in which actions take place and their relevance for human interactions can also affect action execution.

This Research Topic brought together researchers studying socially relevant aspects of cognition (e.g., action observation, imitation, joint and complementary actions) with a wide range of methodologies and theoretical points of view.

Altogether, their contributions carefully represent the status of the field and foresee future developments.

## Shared representations between action and perception

The topic of shared representations between perceived and executed actions is largely present throughout this Research Topic.

Chinellato et al. compared the effects of object-oriented hand actions on motor responses in interactive and non-interactive conditions. Their results showed that a socially relevant condition is quickly taken into account by the motor system, producing an overall slowdown of the motor responses (interference effect). Interestingly, this suggests that the emergence of an interference effect is affected not only by the motor properties of the observed action but also by the available social information.

Letesson et al. approached action observation from the point of view of action priming, i.e., facilitation of motor responses following observation.

Eye- and motion-tracking measures showed that agent's gaze and action kinematics are both contributing to the representation of the action goal, and they do so in a complementary manner. Interestingly, this suggests that—while capable of eliciting motor representations independently—combined gaze and action information produces a more refined action representation.

## Individual, linguistic and cultural differences

The use of experimental paradigms including basic action stimuli and simple motor responses allowed for several possible extensions to diverse samples.

Lewkowicz et al. investigated how individual differences affect the successful detection of social intentions. Individual scores in standard questionnaires of imagery and social cognition were correlated with participants' ability to distinguish between actions with the same motor goal but different intentions (personal vs. social). Data showed that the ability to successfully distinguish personal and social actions by recognizing motor deviants is highly correlated with social cognition scores.

De Stefani et al. incorporated expert athletes' reported “attitude” (competitive vs. cooperative) into a classic action observation design which used cooperative and competitive actions. Their data showed that the expected facilitation of motor responses arose when there was a match between attitude and intended action, but only in the case of cooperative participants. On the contrary, competitive participants were overall faster but without showing significant differences between conditions.

Gianelli et al. extended the investigation of action observation with the use of linguistically described actions and reported the results from experiments in two languages. This cross-linguistic approach showed different motor effects in the two languages. While this study did not include any form of social conditions, a similar paradigm (linguist stimuli + motor responses) could be usefully extended to more interactive contexts.

Manera et al. took a cross-cultural approach by presenting a multilingual database to investigate non-conventional communicative gestures across different languages. The authors created a large set of point-light displays reproducing communicative interactions between two agents and single-agent non-communicative actions. Results from testing this set across seven languages, show that the proposed stimuli were correctly recognized as communicative or individual based on the information available in point-light displays.

The paper by Stapel et al. provided further evidence of the possibilities of extending action the investigation to diverse samples. In this case, they tested infants of various ages (9-, 12-, 15-months old) and adults in an eye-tracking experiment investigating the observer's ability to predict the target of an action based on the velocity of natural object-directed actions. Indeed, the authors showed that, as soon as 15-months of age (but not 9 and 12), infants start using velocity information to predict the outcome of observed actions, similarly to what adults do.

## Experimental settings and stimuli

A crucial aspect of investigating social interactions is the possibility to use controlled yet realistic settings.

Pan and Hamilton extended the investigation of action observation to the use of Virtual Characters (VCs) and sequential hand-arm actions. Comparing the response to VCs actions with the same actions sequence indicated by virtual balls, they showed that participants automatically imitated the actions of the VCs, but not those implied by the virtual balls. VCs might thus be a powerful tool for the study of imitation, providing a richer social context compared to the use of isolated pictures or videos.

Reader and Holmes also questioned the use of video-stimuli when measuring action imitation.

By using a novel motion-tracking paradigm, the authors showed that accuracy was worsened in the case of video stimuli, as compared to face-to-face interaction. Based on these results, the authors suggest that previously reported effects might have been biased by the use of these stimuli and the potential limitations of video stimuli should be further investigated.

## Shared goals, complementary actions

Sacheli et al. reviewed how realistic contexts could integrate a manipulation of the interpersonal cognitive/emotional dimension to investigate the impact of these factors on interacting behaviors. The authors presented and discussed the possibilities of a novel joint-grasping task, which allow for disentangling individual and shared goals in controlled yet naturalist contexts.

Sartori and Betti took a similar approach while reviewing existing evidence on complementary actions, i.e., forms of interaction where agents have to adapt their individual actions to a shared goal, e.g., one individual's action complete the one of the other in order to achieve a common aim.

Finally, Rozzi and Coudé provided an extensive discussion of the neural bases of these processes reporting current anatomic-functional evidence regarding how contextual information could be integrated into an extended motor network. This network might subserve basic social functions, such as allowing two individuals to perform complementary actions toward a shared goal.

## Author contributions

CG and MG discussed and agreed upon the content of this Editorial. CG drafted the manuscript and approved its final version.

### Conflict of interest statement

The authors declare that the research was conducted in the absence of any commercial or financial relationships that could be construed as a potential conflict of interest.

